# Volatile Organic Compound (VOC) Removal by Vapor Permeation at Low VOC Concentrations: Laboratory Scale Results and Modeling for Scale Up

**DOI:** 10.3390/membranes1010080

**Published:** 2011-03-03

**Authors:** Georgette Rebollar-Perez, Emilie Carretier, Nicolas Lesage, Philippe Moulin

**Affiliations:** 1 Université Paul Cézanne Aix Marseille, Laboratoire de Mécanique, Modélisation et Procédés Propres (M2P2—UMR 6181), Europôle de l'Arbois, BP. 80, Bâtiment Laennec, Hall C, 13545 Aix en Provence Cedex 04, France; E-Mails: rebollar@unam.mx (G.R.-P.); emilie.carretier@univ-cezanne.fr (E.C.); 2 TOTAL-Pôle de Recherche et Développement Mont/Lacq, RN 117 BP47, 64000 Lacq, France; E-Mail: nicolas.lesage@total.com

**Keywords:** vapor permeation, PDMS VOC recovery, air treatment

## Abstract

Petroleum transformation industries have applied membrane processes for solvent and hydrocarbon recovery as an economic alternative to reduce their emissions and reuse evaporated components. Separation of the volatile organic compounds (VOCs) (toluene-propylene-butadiene) from air was performed using a poly dimethyl siloxane (PDMS)/α-alumina membrane. The experimental set-up followed the constant pressure/variable flow set-up and was operated at ∼21 °C. The membrane is held in a stainless steel module and has a separation area of 55 × 10^−4^ m^2^. Feed stream was set to atmospheric pressure and permeate side to vacuum between 3 and 5 mbar. To determine the performance of the module, the removed fraction of VOC was analyzed by Gas Chromatography/Flame Ionization Detector (GC/FID). The separation of the binary, ternary and quaternary hydrocarbon mixtures from air was performed at different flow rates and more especially at low concentrations. The permeate flux, permeance, enrichment factor, separation efficiency and the recovery extent of the membrane were determined as a function of these operating conditions. The permeability coefficients and the permeate flux through the composite PDMS-alumina membrane follow the order given by the Hildebrand parameter: toluene > 1,3-butadiene > propylene. The simulated data for the binary VOC/air mixtures showed fairly good agreement with the experimental results in the case of 1,3-butadiene and propylene. The discrepancies observed for toluene permeation could be minimized by taking into account the effects of the porous support and an influence of the concentration polarization. Finally, the installation of a 0.02 m^2^ membrane module would reduce 95% of the VOC content introduced at real concentration conditions used in the oil industry.

## 1. Introduction

Volatile organic compounds (VOCs) are involved in atmospheric pollution and green house effect. Some of these compounds might be recovered, instead of being released to the atmosphere, by several methods such as condensation, absorption, adsorption, *etc.* Among these processes, vapor permeation has several advantages since it requires compact equipment, it is non destructive and it is not energy-intensive. Over the past ten years, vapor permeation has been proven to be a feasible alternative to conventional processes in the recovery of several halogenated VOCs and monomers [1]. In recent years, this process has found other applications such as in the recovery of hydrocarbon VOCs from the petroleum industry facilities; these applications are still under development [2].

Within the membranes used for the recovery of volatile organic compounds, composite membranes offer several advantages over other kinds. They are composed of a selective, defect-free layer that performs the vapor separation while another porous layer gives mechanical strength. Poly dimethyl siloxane (PDMS) is one of the most used polymers as selective permeation layer. It can be easily fabricated and thus is readily available for its use on large scales. The use of dimensionless solubility parameters showed that PDMS has good selectivities towards a wide variety of VOCs (e.g., hydrocarbons).

The recovery of toluene, propylene and 1,3-butadiene, which are compounds of particular concern in the petroleum industry, is focused on in this study. Since several petroleum activities such as oil storage or distribution, emit pollutants at low flow rates and variable concentrations, vapor permeation appears to provide a flexible recovery solution.

The low concentration VOC recovery from binary, ternary or quaternary mixtures is studied here. Some phenomenological relations between the permeate flux, the VOC concentrations and the flow velocity of polluted air have been calculated from lab scale results. The experimental results were finally compared to those obtained with a simplified mathematical model built on the basis of a simple piston flow model, which provides a conservative estimate on the separation.

## 2. Results and Discussion

### 2.1. VOC and Membrane

Toluene anhydride, (Tol, 99.5% SDS, France), 1,3-butadiene (BD, Alpha gaz, 99.6% Air Liquide) and propylene (Prop, Alpha gaz, 99.5% Air Liquide, France) were the VOC used in the vapor permeation experiments.

The composite membrane was manufactured by Pervatech (The Netherlands). It is a microporous ceramic support having a thin PDMS layer coated on the internal surface of the tube. These characteristics provided an effective membrane area of 55 × 10^−4^ m^2^. The membrane is held in a cartridge consisting of a simple shell and tube design built with a stainless steel module containing one tube membrane inside.

### 2.2. Set-up

**Figure 1:**
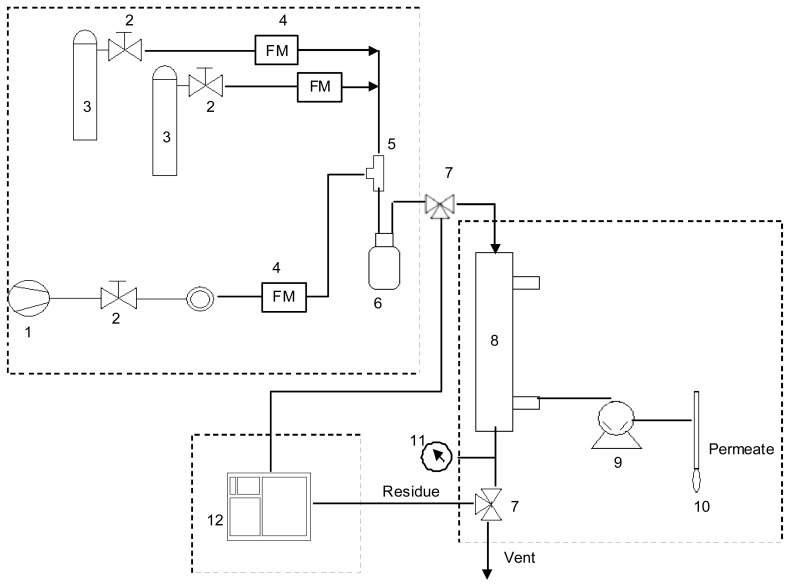
Schematic diagram of the experimental set up: (1) compressed air, (2) regulating valves, (3) gas cylinders, (4) flow meters (FM), (5) T connector, (6) mixing flask, (7) three way valves, (8) membrane module, (9) vacuum pump, (10) bubble flow meter, (11) pressure indicator, (12) gas chromatography equipment.

The experimental set-up of the single membrane module is a standard permeation design, shown in Figure 1. It is comprised of three sections: mixture generation, membrane module and on line gas chromatographic analysis. The feed gas mixture at a given composition was obtained by introducing controlled flows of dried air (1) and VOC gases (3). The actual composition of the gas mixture was determined by gas chromatography (12). The membrane cartridge (8) was installed under a hood at room temperature (23 ± 2 °C). The feed gas was introduced at atmospheric pressure. The VOC permeation is induced by keeping the permeate pressure lower than the feed gas pressure. Vacuum is obtained using an Ilmvac MP 601 Tp vacuum pump (9) (Fisher Bioblock) and is measured with a pirani DN 16 captor adapted to a manual vacuometer (Thyraconte VD84M, Fisher Bioblock). Vacuum pressure was maintained between 3 and 5 mbar (300 and 500 Pa).

### 2.3. Chromatographic Analysis

The different lines were analyzed by gas chromatography (GC) (Varian GC-3800, Varian France, S.A.). The apparatus was equipped with a Plot alumina/KCl column and with an FID detector to know the actual VOC concentrations in the gaseous phase. Plot columns are well suited for hydrocarbons analysis [3]. A six port selection valve connected to the injector port of the GC apparatus allows the analysis of the system inlet and outlet lines. This valve is heated to 80 °C. The injection port temperature is maintained at 200 °C and injections are made in a split-splitless mode. The sampling loop has a volume of 250 μL. The column oven was programmed as follows: initial temperature, 100 °C for 0.5 min; temperature increase up to 180 °C at 20 °C/min and maintained for 2.5 min. The detector temperature is set at 250 °C. Helium is chosen as a vector gas.

### 2.4. Generation of Gaseous Mixtures

Different binary (VOC/air), ternary (two VOC in air) and quaternary (three VOC in air) mixtures were generated at different concentrations and flow rates. Relatively low concentrations are particularly analyzed within the small range studied, from 0.14 up to 7.3 mol%. The gas mixtures were generated as follows: the inlet air streams were dehydrated by calcium sulfate and regulated by a gas flow meter [4]. Dry air was mixed with known quantities of the model VOC chosen. Liquid toluene was injected continuously with a syringe inserted in the tube and was vaporized by the incoming dry air. A mixing flask was installed before the membrane module to homogenize the prepared mixture. No toluene condensation was observed throughout the whole study. For mixtures generated with air, 1,3-butadiene and propylene, the VOC gas introduction was regulated by a manometer installed in each gas cylinder, controlling the flow rates by direct reading scale gas flow meters (Sigma Aldrich). The VOC gas flow rates were varied between 0.2 and 40 mL·min^−1^. For all the generated gas mixtures, the stability of each VOC feed concentration was verified by gas chromatography. In all cases, the permeation through the membrane was achieved by pressure difference connecting a vacuum pump at the permeate side, keeping low vacuum (3–5 mbar or 300–500 Pa).

Binary mixtures (VOC/air) were analyzed at three different flow rates: 220, 330 and 550 mL·min^−1^ for toluene, and 330, 550 and 880 mL·min^−1^ for 1,3-butadiene and propylene. Ternary mixtures (two VOC in air) and quaternary mixtures (the three model VOC in air) were only analyzed at a flow rate of 550 mL·min^−1^.

## 3. Laboratory Scale Results

**Figure 2:**
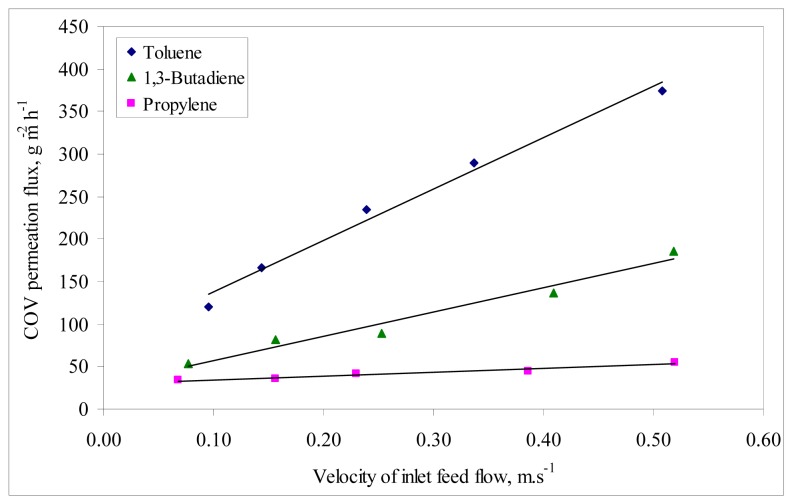
Effect of inlet feed flow rate on permeation flux at constant feed molar fraction: 1.5% for toluene and 1.7% for 1,3-butadiene and propylene.

The influence of the feed flow rate on the permeance behavior of the VOC/air mixtures was analyzed by varying the feed flow rate from 150 to 1200 mL·min^−1^. The equivalent velocities across the longitudinal membrane section ranged between 0.096 and 0.52 m·s^−1^. The VOC feed molar fraction was kept constant at 1.47% for toluene and at 1.7% for 1,3-butadiene and propylene. The variation of the VOC permeation flux is represented as a function of the velocity of feed mixtures in Figure 2. The permeate flux increases with feed velocity and is highest at the highest velocity of the mixtures in the module following always the order toluene > 1,3-butadiene > propylene. At the highest mixture velocity circulating in the module, the permeation fluxes are 375 g·m^−2^·h^−1^ for toluene, 185 g·m^−2^·h^−1^ for 1,3-butadiene and 55 g·m^−2^·h^−1^ for propylene.

**Figure 3:**
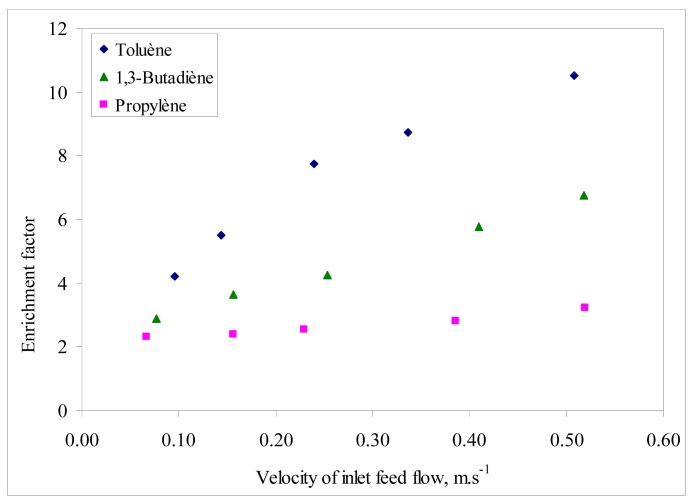
Effect of feed velocity on enrichment factor at constant feed molar fraction: 1.5% for toluene and 1.7% for 1,3-butadiene and propylene.

The enrichment factor of the VOC/air binary mixtures was also analyzed as a function of feed velocity and the results are presented in Figure 3. For a given component *i*, the enrichment factor is defined as the ratio between its concentrations on the feed (F,i) and the permeate (P,i) (*i.e.*, C_F,i_/C_P,i_) [5]. The trends of enrichment factor for the VOC analyzed followed a similar increasing behavior with feed velocity, as for permeation flux. The enrichment factor is highest for toluene in the operating conditions interval in agreement with the affinity order between the selective layer and the VOC as mentioned earlier.

Merkel *et al.* [6] found that permeability through PDMS is more influenced by the solubility coefficient than by the diffusivity coefficient because the latter changes less, so the more soluble penetrant compounds are the most permeable. Thus, VOC permeability in PDMS is largely determined by its relative solubility. According to several studies on VOC permeation through PDMS membranes, the more condensable compounds generally show higher permeabilities, because of higher solubility into the polymer [7-9]. Thus, these two factors contributed to the observed permeation fluxes and enrichment factors from Figure 2 and Figure 3.

### 3.4. Phenomenological Relations to Estimate the Permeate Flux

The results regarding the VOC permeation behavior at constant feed flow rate and inlet VOC concentration from the VOC/air mixtures allowed the determination of the phenomenological relations that describe the permeation flux. These relations take the general following form

(1) $$\text{J}\;=\text{a}\;{\text{v}}^{\text{b}}{\text{C}}^{\text{c}}$$

where J is the permeation flux (g·m^−2^·h^−1^), *v* is the incoming velocity of the gaseous mixture (m·s^−1^), *C* is the VOC concentration (g·m^−3^) in the binary mixture with air and *a, b* and *c* are coefficients obtained from the experimental data. The corresponding relations for toluene, 1,3-butadiene and propylene are given in Table 1. These expressions are valid for a molar concentration interval between 0.1 and 7.5% and for laminar flow regimes (Re < 300).

**Table 1:** Correlations of predicted flux obtained from experimental data.

**VOC**	**Correlation for predicted flux**
Toluene	J = 10.4·C^1.03^·v^0.68^
1,3-Butadiene	J = 12.2·C^0.89^·v^0.62^
Propylene	J= 0.83·C^1.42^·v^0.69^

Note: J is the predicted flux; C is the concentration (g·m^−3^); and v is the inlet gas velocity (m·s^−1^).

## 4. Modeling and Scale Up

Models are mathematical tools that are helpful in determining the operating conditions that could maximize the performance of a system. The mathematical model used to represent the behavior of the present vapor permeation process was developed based on the experimental data obtained throughout this study. In fact, only the binary VOC/air mixtures were represented by the model. The model could allow the sizing of a larger permeation unit to treat streams at real emission conditions.

**Figure 4:**
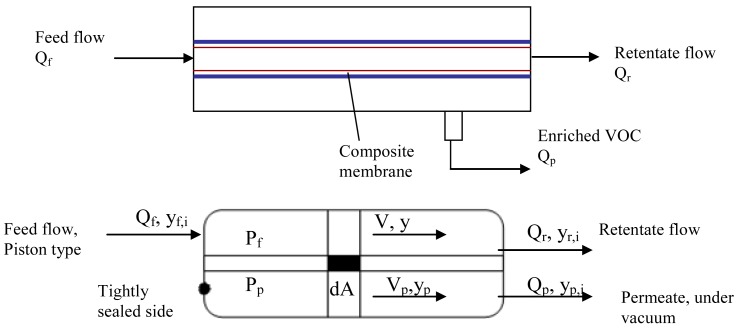
Schematic diagram of the membrane process (top) and differential unit of the membrane (bottom) for the volatile organic compound (VOC) separation based on piston flow model.

The potential separation performance of the membrane process was evaluated on the basis of a simple piston flow model, which provides a conservative estimate on the separation [10]. This model could also be described as considering concentration changes along the membrane in small steps. The model proposed in the present work was solved using two hundred steps over the internal surface of the ceramic tube, provided that the selective membrane is coated on this side. Figure 4 illustrates the membrane separation using the piston flow configuration, where the pressure variation along both feed and permeate sides are assumed to be negligible.

The following relations can be formulated based on permeation and mass balance equations to describe the permeation process [11]:

(2) $$-{\text{y}}_{\text{p}}{\text{dQ}}_{\text{f}}=\Pi_{\text{VOC}}({\text{P}}_{\text{f}}{\text{y}}_{\text{f}}-{\text{P}}_{\text{p}}{\text{y}}_{\text{p}})\text{dA}$$

(3) $$-(1-{\text{y}}_{\text{p}}){\text{dQ}}_{\text{f}}=\Pi_{\text{air}}[{\text{P}}_{\text{f}}(1-{\text{y}}_{\text{f}})-{\text{P}}_{\text{p}}(1-{\text{y}}_{\text{p}})]\text{dA}$$

(4) $$\text{d}({\text{Q}}_{\text{f}}{\text{y}}_{\text{f}})={\text{y}}_{\text{p}}{\text{dQ}}_{\text{f}}$$

where y_f_ and y_p_ are the VOC molar compositions of the feed and permeate sides of the membrane, Q_f_ is the molar gas flow rate (mol·s^−1^) on the feed side, A is the membrane area and Π_i_ is the gas compound permeance.

Developing the equations for a differential element of the membrane, a system of two differential equations is obtained:

(5) $$\text{dV}/\text{dA}=-[\Pi_{\text{VOC}}({\text{y}}_{\text{f}}\text{P}-{\text{y}}_{\text{p}}\text{P})+\Pi_{\text{air}}[(1-{\text{y}}_{\text{f}})\text{P}-(1-{\text{y}}_{\text{p}})\text{P}]]$$

(6) $$\text{dV}/\text{dA}={\text{y}}_{\text{f}}/\text{V}[\Pi_{\text{VOC}}({\text{y}}_{\text{f}}{\text{P}}_{\text{f}}-{\text{y}}_{\text{p}}{\text{P}}_{\text{p}})+\Pi_{\text{air}}[(1-{\text{y}}_{\text{f}}){\text{P}}_{\text{f}}-(1-{\text{y}}_{\text{p}})\text{P}]]-\Pi_{\text{VOC}}({\text{y}}_{\text{f}}\text{P}-{\text{y}}_{\text{p}}{\text{P}}_{\text{p}})/\text{V}$$

In these expressions, V is the binary mixture molar flow rate (mol.s^−1^), A is the total membrane surface (55 × 10^−4^ m^2^), P_f_ is the feed pressure (10^5^ Pa), P_p_ is the permeate pressure in the module (vacuum, 500 Pa), and Π_i_ represents the permeance of each VOC or air (mol·m^−2^·s^−1^·Pa^−1^). The ordinary differential equations system represented by relations (5) and (6) was solved by finite differences using Matlab R2007a (V 7.4.0.287). The pure gases experimental values for each compound were introduced in the equations system. The molar fractions were varied in the interval studied for the binary mixtures permeation. The limit conditions to solve the system were chosen as follows: A = 0, y = y_f_ and V = Q_f_.

**Figure 5:**
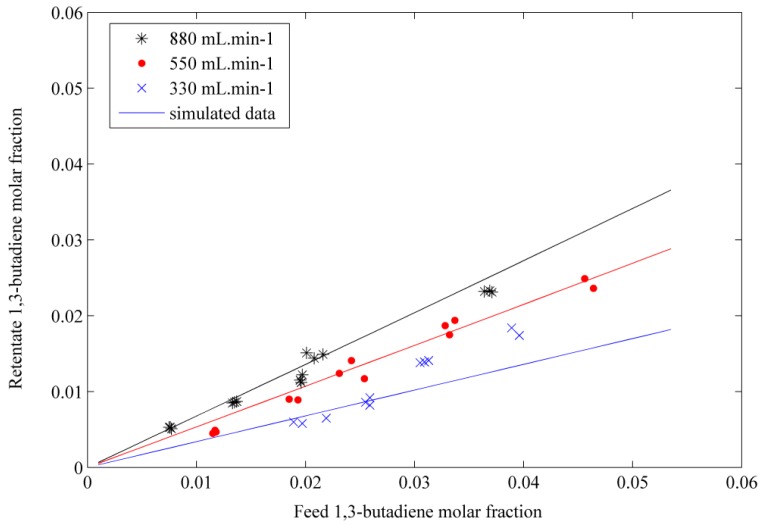
Variation of 1,3-butadiene molar fraction in retentate as a function of its molar fraction in feed at three different flow rates. The symbols represent the experimental data; the straight lines correspond to the simulation results.

The simulated results obtained by solving the differential equations system are compared with the experimental data of the binary mixtures separations. The molar fraction in the retentate was represented by the model as a function of the feed molar fraction for each VOC species. The results are shown in Figure 5 for butadiene. The results obtained by the model, represented by the solid lines, show that there is fairly good agreement with the experimental data for these two species. The model can represent the permeation behavior of the binary mixtures. The permeability coefficients obtained at the beginning of the study hold then true for 1,3-butadiene and propylene.

A discrepancy was observed when the model was applied to study the permeation of binary toluene. The simulated results were underestimated with respect to the experimental data. The permeance value for toluene was taken from the literature [13] and was introduced in the equations system. The permeance of pure toluene was determined by pervaporation, introducing the liquid compound into the membrane module. The toluene permeance was thus approximated by taking a value from the experimental data concerning the binary toluene/air permeation. Values from the most concentrated toluene mixtures were tested. A permeance of 6.5 × 10^−7^ mol·m^−2^·s^−1^·Pa^−1^, being inferior to that given by Heymes [13], was thus obtained and introduced to the model. The simulated retentate molar fractions as a function of the feed molar fraction were of the same order of magnitude compared to experimental data. It seemed that a better agreement was obtained with this new and approximated toluene permeance value. However, further studies taking into account the effects of the porous support and an influence of the concentration polarization could give a better adjustment of the model to the experimental results.

### 3.9. Determination of the Membrane Surface in the Permeation of a Mixture under Real Conditions

In this section, the variation of the VOC molar fraction in the retentate was analyzed as a function of the membrane surface for a mixture introduced at real emission concentrations. The binary mixtures for each VOC were simulated by introducing to the model the values of their average molar fractions corresponding to the real concentrations emitted *in situ*. The temperature and flow rate conditions were kept the same as for binary mixtures under laboratory conditions.

**Figure 6:**
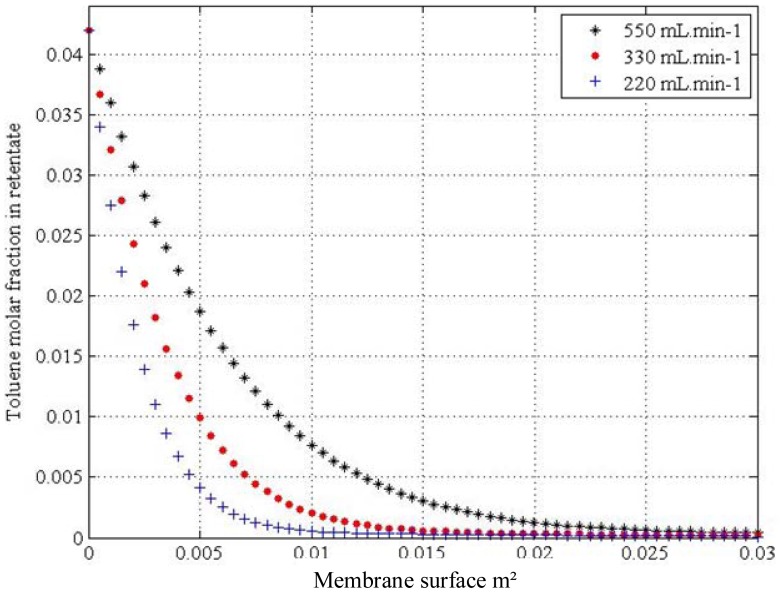
Variation of the toluene molar in retentate as a function of the membrane surface at three different flow rates. Upstream pressure, 1 bar (10^5^ Pa); downstream pressure, 5 mbar (500 Pa).

Figure 6 shows the simulated results for the case of toluene permeation. The profiles are obtained at the three flow rates chosen in Section 2.5. From these profiles it can be seen that the membrane surface required to achieve the abatement of the VOC depends on the mixture flow rate. Thus, the abatement of 95% toluene in binary mixture would require a membrane surface of 6.5 × 10^−3^ m^2^ introducing the feed at 220 mL·min^−1^, of 10 × 10^−3^ m^2^ at a flow rate of 330 mL·min^−1^ and of 17.5 × 10^−3^ m^2^ at 550 mL·min^−1^.

A similar analysis for the other two VOCs, 1,3-butadiene and propylene, also gives the necessary membrane surface to achieve an abatement of 95% of their feed concentration. The feed concentrations for toluene, propylene and butadiene introduced in the model correspond to the average concentrations of the industrial case. The feed flow rates introduced in the model corresponded to those studied for the binary mixtures under laboratory conditions. Thus, introducing the average molar fraction of 7 × 10^−5^ for 1,3-butadiene, the required membrane would be 0.013 m^2^ at 330 mL·min^−1^, 0.022 m^2^ at 550 mL·min^−1^ and 0.035 m^2^ at 880 mL·min^−1^. For the case of propylene, introducing the average molar fraction of 3.5 × 10^−6^, the membrane surface required to abate 95% this inlet concentration would be 0.027 m^2^ given a feed flow rate of 330 mL·min^−1^, 0.044 m^2^ for a feed flow rate of 550 mL·min^−1^, and 0.07 m^2^ given a feed flow rate of 880 mL·min^−1^.

Ternary and quaternary mixtures were also evaluated introducing the VOC at real concentration conditions. Larger commercial membrane modules, with characteristics similar to those of the membrane used in the present study, are readily available in the market.

Emitting the hypothesis that the VOC permeation in quaternary mixture follows the behavior of the pure gases permeation observed in this study, the installation of a 0.02 m^2^ module would assure the abatement of 95% the VOC introduced at real concentration conditions at a low feed flow rate, between 220 and 330 ml·min^−1^. The final module characteristics and membrane surface will depend on the specific requirements of the treated streams and the total investment costs.

## 5. Conclusion

For the binary mixtures, the permeate flux of the three studied VOCs increased with concentration and feed velocity (and flow rate) under the conditions of temperature and pressure tested. Phenomenological relations were obtained to predict permeate flux as a function of the velocity and inlet concentration. The enrichment factor for binary VOC mixtures also increased with increasing feed velocity. The coupling effects were studied and the results for ternary and quaternary mixtures are within a variation of 15% from the binary mixtures.

The modeling of the results for the binary VOC/air mixtures showed fairly good agreement with the experimental results in the case of 1,3-butadiene and propylene. The discrepancies observed for toluene permeation could be minimized by taking into account the effects of the porous support and the influence of the concentration polarization. Finally, the installation of a 0.02 m^2^ membrane module would reduce 95% of the VOC content introduced at real concentration conditions used in the petroleum industry.
